# Cognitive Functions and Neurodevelopmental Disorders Involving the Prefrontal Cortex and Mediodorsal Thalamus

**DOI:** 10.3389/fnins.2018.00033

**Published:** 2018-02-06

**Authors:** Zakaria Ouhaz, Hugo Fleming, Anna S. Mitchell

**Affiliations:** Department of Experimental Psychology, University of Oxford, Oxford, United Kingdom

**Keywords:** autism, decision-making, dopamine, glutamate, learning, olfaction, working memory, schizophrenia

## Abstract

The mediodorsal nucleus of the thalamus (MD) has been implicated in executive functions (such as planning, cognitive control, working memory, and decision-making) because of its significant interconnectivity with the prefrontal cortex (PFC). Yet, whilst the roles of the PFC have been extensively studied, how the MD contributes to these cognitive functions remains relatively unclear. Recently, causal evidence in monkeys has demonstrated that in everyday tasks involving rapid updating (e.g., while learning something new, making decisions, or planning the next move), the MD and frontal cortex are working in close partnership. Furthermore, researchers studying the MD in rodents have been able to probe the underlying mechanisms of this relationship to give greater insights into how the frontal cortex and MD might interact during the performance of these essential tasks. This review summarizes the circuitry and known neuromodulators of the MD, and considers the most recent behavioral, cognitive, and neurophysiological studies conducted in monkeys and rodents; in total, this evidence demonstrates that MD makes a critical contribution to cognitive functions. We propose that communication occurs between the MD and the frontal cortex in an ongoing, fluid manner during *rapid* cognitive operations, via the means of efference copies of messages passed through transthalamic routes; the conductance of these messages may be modulated by other brain structures interconnected to the MD. This is similar to the way in which other thalamic structures have been suggested to carry out forward modeling associated with rapid motor responding and visual processing. Given this, and the marked thalamic pathophysiology now identified in many neuropsychiatric disorders, we suggest that changes in the different subdivisions of the MD and their interconnections with the cortex could plausibly give rise to a number of the otherwise disparate symptoms (including changes to olfaction and cognitive functioning) that are associated with many different neuropsychiatric disorders. In particular, we will focus here on the cognitive symptoms of schizophrenia and suggest testable hypotheses about how changes to MD-frontal cortex interactions may affect cognitive processes in this disorder.

## Introduction

Typically, prefrontal cortex (PFC) is associated with the domain of executive functions. In recent years, however, causal evidence has emerged that the mediodorsal nucleus of the thalamus (MD) also constitutes a key structure in various cognitive processes, in particular during rapid integration of new learning, working memory, and adaptive decision-making in primates and rodents (Sommer and Wurtz, 2002, 2004a,b, 2006, 2008; Mitchell et al., 2007a,b, 2014; Cross et al., 2013; Mitchell and Chakraborty, 2013; Parnaudeau et al., 2013, 2015; Browning et al., 2015; Ouhaz et al., 2015, 2017; Chakraborty et al., 2016; Bolkan et al., 2017; Schmitt et al., 2017). We now provide a review of the evidence to show that the MD works in partnership with the frontal cortex, in addition to other brain structures, during cognitive processes. Our understanding of the functioning of the thalamus has been re-evaluated following the proposal of Guillery and Sherman (Sherman and Guillery, 2001, 2002, 2013; Guillery and Sherman, 2002; Sherman, 2004) that the thalamus has an ongoing, interactive relationship with the cortex.

Amongst a number of important developments is the identification of several “higher-order” thalamic nuclei (including, of particular relevance to this review, the MD), which are so called because the “driver” message they receive comes from layer 5 of the cortex, rather than from primary sensory structures (Sherman and Guillery, 2013). These cortico-thalamic inputs appear, for the most part, to be branches of corticofugal axons that also project to subcortical motor structures (e.g., superior colliculus, claustrum, zona incerta, basal ganglia), and therefore the message conveyed via higher order thalamic nuclei, namely via the transthalamic route, might best be described as a copy of an efferent motor command (Guillery, 1995, 2005). This efference copy (or corollary discharge) of an already processed cortical message is then relayed on to a different region of cortex for further processing.

It has been suggested that the function of these higher order nuclei, which may include some of the subdivisions of the mediodorsal thalamus (see below), as well as subdivisions of the pulvinar and lateral posterior nucleus (amongst others) is, at least in part, to inform other regions of the cortex about upcoming motor commands (Sherman, 2007), thus establishing a forward predictive model of expectations about actual motor responses. We will review recent causal evidence from monkey lesions studies involving the magnocellular subdivision of the MD (MDmc) to show that this may indeed be so in relation to cognitive functions too. We will also consider recent rodent animal studies involving optogenetics and neurophysiology that demonstrate that MD thalamic mechanisms may contribute to cognitive functions carried out by the cortex. Lastly, we will review the evidence from rodent studies indicating how early onset damage to the MD, resulting in changes to the frontal cortex, may help to account for some of the behavioral and cognitive deficits associated with schizophrenia: briefly, it has been proposed elsewhere that some of the sensory psychotic symptoms (e.g., auditory hallucinations and delusions) of schizophrenia could be accounted for by disruption of the efference copying process in the cortex, resulting in an inability to take account of self-generated actions during perceptual processing (Frith, 2005). We will reconsider this model in light of the suggestion that the thalamus contributes to forward modeling, extend this discussion to encompass the cognitive symptoms of schizophrenia as well, and suggest ways in which animal models could shed more light on the importance of the transthalamic route of information processing for cognitive functions too. The argument that will be advanced in this paper is that transthalamic pathophysiology, affecting in particular the higher order nuclei such as MD, may constitute an early feature of several neurodevelopmental disorders, and that this may help to account for some of the behavioral and cognitive symptoms associated with them. Furthermore, while many studies still need to be completed to verify this, we will propose that the profile of the MD must be elevated so that it may be considered as a potential target for treatment options in such diseases.

## Neuroanatomy of higher order thalamic nuclei—mediodorsal thalamus

**Figure 1:**
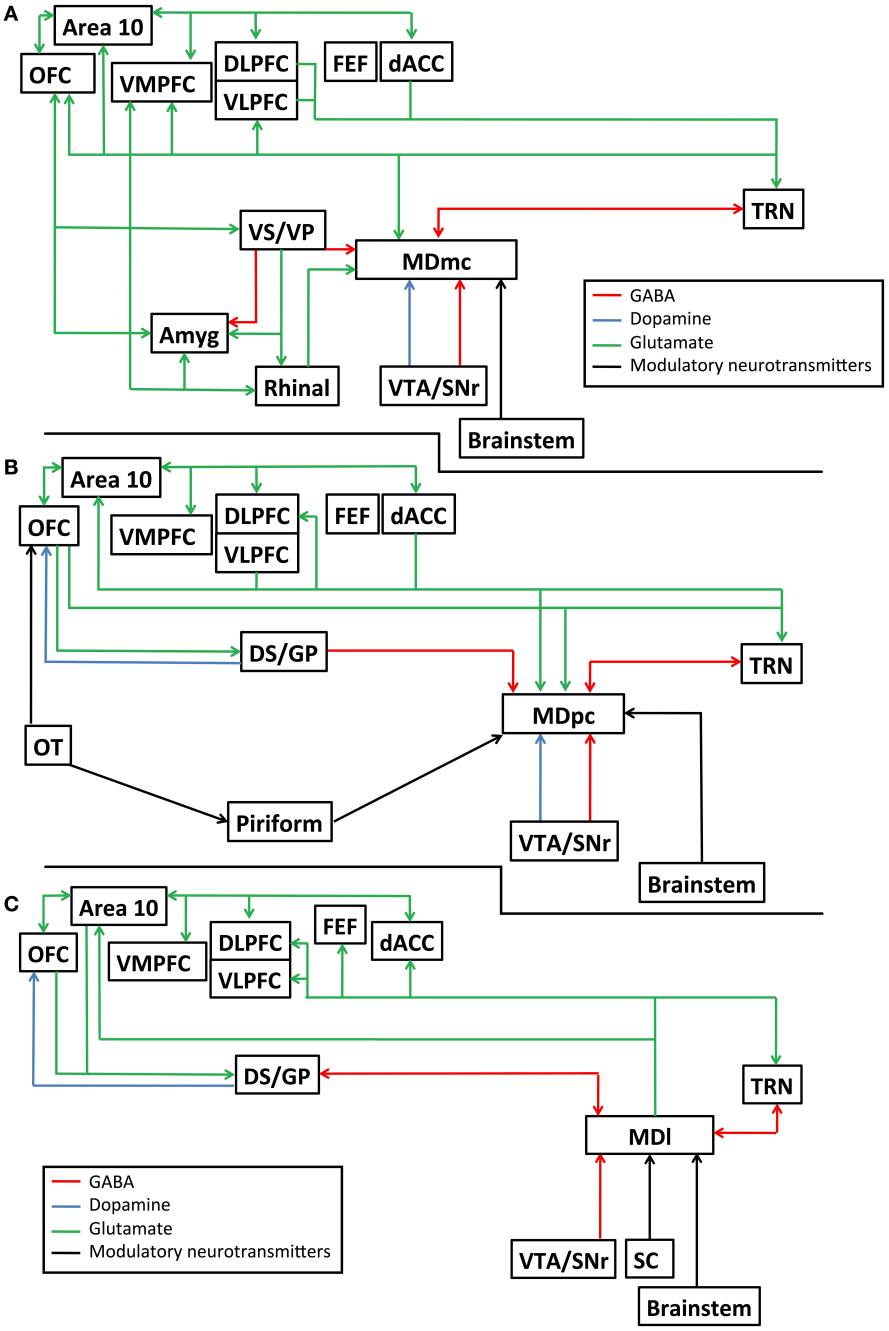
Schematic diagram detailing the known interconnections, neurotransmitters and neuromodulatory pathways connecting the mediodorsal thalamus and other brain structures, **(A)** for the magnocelluar subdivision of the mediodorsal thalamus (MDmc); **(B)** for the parvocelluar subdivision of the mediodorsal thalamus (MDpc); and **(C)** the lateral subdivision of the mediodorsal thalamus (MDl). Amyg, amygdala; Area 10, frontopolar cortex, Brodmann area 10; dACC, dorsal anterior cingulate cortex; DLPFC, dorsolateral prefrontal cortex; DS/GP, dorsal striatum/ globus pallidus; FEF, frontal eye fields; OFC, orbitofrontal cortex; OT, olfactory tubercle; Piriform, piriform cortex; Rhinal, perirhinal and entorhinal cortex combined; TRN, reticular thalamic nucleus; SC, superior colliculus; VLPFC, ventrolateral prefrontal cortex; VMPFC, ventromedial prefrontal cortex; VS/VP, ventral striatum/ ventral pallidum; VTA/SNr, ventral tegmental area/substantia nigra pars reticulata.

The primate MD can be divided based on its cell morphology into at least four distinct subdivisions, namely the magnocellular MD (MDmc), the parvocellular MD (MDpc), the caudodorsal MD (MDcd), and the lateral MD (MDl). The rodent MD has at least three subdivisions: medial MD, central MD, and the lateral MD, that overlap with the above subdivisions. The major interconnections of these primate subdivisions are unique to each subdivision and have been extensively reviewed (Mitchell and Chakraborty, 2013; Mitchell, 2015) and are further detailed in Figures 1A–C. In Figures 1A–C, we also highlight the known neurotransmitters and neuromodulators of these interconnections to help provide a greater awareness of the divergent inputs and outputs associated with these nuclei that are potentially involved in transthalamic routes of communication. Briefly, and in general, the different subdivisions of the MD thalamus each have unique efferent and afferent connections with the prefrontal cortex, cingulate cortex, insular cortex, supplementary motor cortex, reticular thalamus, basal ganglia, and output structures of the pallidum. In addition, parts of the medial temporal lobes (namely the perirhinal and entorhinal cortex and the amygdala) send projections to the MDmc. The connections between the cortex and MD are all glutamatergic while the MD connections received from the reticular thalamus and pallidum are GABAergic. Finally, selective neuromodulatory inputs (e.g., dopamine, norepinephrine, serotonin, acetylcholine, and noradrenaline), originating from different structures in the midbrain and in the brainstem project selectively either to the MDmc, the MDpc, the MDcd, or the MDl. Thus, given this known neuroanatomy, each individual MD subdivision must be considered as an interdependent nucleus that is distinct to its neighbors in its function and in its contribution to cognitive processes.

All thalamic nuclei, including the different subdivisions of the MD, receive afferents from cortical layer 6 (that are transmitted via the reticular thalamus), while only some nuclei, namely the higher order thalamic nuclei, which includes some of the subdivisions of the MD, also receive cortical afferents from layer 5 (corticothalamic inputs), in the form of a collateral projection (efference copy) going to a motor output structure (Gilbert and Kelly, 1975; Abramson and Chalupa, 1985; Ono and Niimi, 1986; Giguere and Goldman-Rakic, 1988; Groenewegen, 1988; Schwartz et al., 1991; Kuroda et al., 1992a,b; Ojima, 1994; Bourassa and Deschênes, 1995; Bourassa et al., 1995; Xiao et al., 2009; Timbie and Barbas, 2015). Sherman and Guillery (1998) proposed that the corticothalamic inputs from layer 5 are “drivers,” whilst those from layer 6, which can also be reciprocal, are “modulators.”

This classification of drivers and modulators has been based on distinct neuroanatomical differences between the two types of corticothalamic axon observed in the visual system and somatosensory (barrel) cortex (Guillery, 1995; Rockland, 1998; Rouiller and Welker, 2000; Sherman and Guillery, 2001; Guillery and Sherman, 2002; Sumser et al., 2017). Xiao et al. (2009) and Schwartz et al. (1991) have identified different clusters of PFC cortical projections (i.e., from layer 5 and from layer 6) going to different subdivisions of the MD. Briefly, drivers have large boutons, contact proximal dendritic segments (Feig and Harting, 1998; Guillery et al., 2001) and so are able to determine the receptive field properties of the thalamic relay cell (Guillery, 1995; Sherman and Guillery, 1998), whereas modulators (such as layer 6 axons) have small boutons that contact peripheral dendritic segments and so they do not determine the receptive field, but instead modify the way in which signals are transmitted through the thalamus, via the thalamic relay cells (Sherman and Guillery, 2013).

Given that it is now widely recognized that distributed and interdependent neural networks support all of our daily activities and cognitive abilities, the source of the driver inputs to these neural networks will help to determine its function and if a given thalamic nucleus is classified as higher order (Guillery, 1995; Sherman and Guillery, 1996, 1998, 2013). Further, the source of the driver input will help to identify its functional contribution to these neural networks that support higher cognitive processes. The “first order thalamic relays” receive their driver input from peripheral sensory neurons/organs via ascending pathways (e.g., lateral geniculate nucleus is a first order thalamic relay of driver information from the retina; Guillery, 1995), and this represents the first relay of a given type of information to cortex. By contrast, the “higher order thalamic nuclei” (such as some subdivisions of the MD (see further details below), lateral posterior nucleus or pulvinar) receive very little, if any, sensory inputs. Instead, it has been proposed that they receive their driver input from layer 5 of the cortex itself (Reichova and Sherman, 2004). Unlike the layer 6 input, however, this layer 5 collateral input is not a feedback projection (Van Horn and Sherman, 2004), and instead is presumed to be feedforward, capable of transmitting already processed cortical information on to other cortical areas.

**Figure 2:**
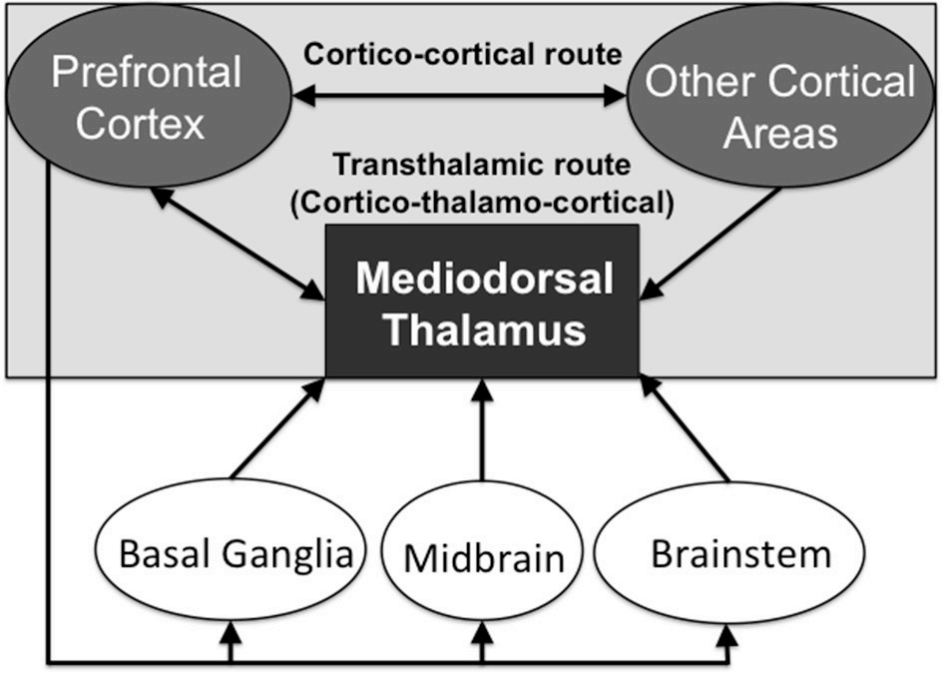
Schematic diagram showing the direct cortico-cortical routes of neural transmission and the indirect transthalamic (cortico-thalamo-cortical) routes of transmission, via the higher order thalamic nuclei. Neuromodulatory inputs from other interconnected brain structures regulate the transmission via the transthalamic route.

This higher order relay classification is also linked to the “core-like” hypothesis of cortico-thalamo-cortical circuitry (Jones, 2002). The recent work of Kuramoto et al. (2017) documents that 13 out of 14 MD neurons, which they juxtacellularly labeled, showed dispersed projections to widespread distributed frontal cortical areas and layers (suggesting “core-like” thalamic neurons were labeled: although according Jones, 2002 “core-like” neurons project to cortical layer 4 only). In contrast, only 1 out of the 14 neurons selectively projected to Layer 1 (suggesting a “matrix-like” thalamic neuron). For a discussion about the “core-matrix” hypothesis of thalamocortical relay cells, see Jones (2007). These thalamic relay cells identified in the different subdivisions of the MD are potentially able to aid in cortico-cortical transmission, in addition to the cortico-cortical direct connections (see Figure 2). By this reasoning, these higher order nuclei can be viewed as an essential link in a transthalamic route that supports information processing across distributed networks of cortical regions.

An important proviso is that all thalamic nuclei also receive other neuromodulatory afferents. These additional neuromodulatory inputs received by either a first order or higher order thalamic nucleus help to influence the transmission to the cortex of its driver signal; these modulators are received from thalamic interneurons, the reticular thalamus, and other structures of the forebrain, midbrain and the brainstem (Jones, 1985; Sherman and Guillery, 1996, 2002; Guillery and Sherman, 2002; Rovó et al., 2012; Halassa and Acsády, 2016).

The fact that MD, as a higher order nucleus, receives dual corticothalamic inputs originating from both layers 5 and 6 of the PFC implies that there are important functional differences in the way in which the MD interacts with the cortex, compared with other, first-order thalamic nuclei which only receive a single cortico-thalamic (layer 6) input (Schwartz et al., 1991). Xiao et al. (2009) showed that about 20% of the PFC layer 5 projections terminate in the MD, although they did not distinguish differences in where their identified cortical neurons terminated on the dendrite sites; these afferents originate mainly from the dorsal and medial PFC areas and go to distinct subdivisions of the MD (namely MDmc and MDpc). In addition to these “driver” inputs, MD also receives additional inputs from many other brain structures, including the medial temporal lobes, the pallidum, the reticular thalamus, MD interneurons (in primates only), midbrain and brainstem, all of which are summarized in Figures 1A–C, where more specific details can be found (Ono and Niimi, 1985; Kuroda and Price, 1991a,b; Ray et al., 1992; Sherman and Guillery, 1996; Mitchell, 2015). All of these other inputs, rather than providing a message for relay by themselves, are proposed to influence whether the driver signals received from the cortex (corticothalamic inputs) get relayed back to the cortex (thalamocortical inputs), via this transthalamic route through the MD. Note that these transthalamic routes connect regions of the cortex that also have direct cortico-cortical interconnections (Figure 2); assuming that there is not a duplication of function here, understanding the different roles that these two pathways play is a question which we are still yet to answer satisfactorily.

Consequently, asking what signals are primarily responsible for driving thalamic relay neurons, how other signals are modulating these neurons, and investigating what messages these higher order thalamic neurons are transmitting to influence the cortex is critical to understanding how the cortex is functioning (Sherman and Guillery, 2013; Guillery, 2017). In this review we are particularly interested in higher cognitive functions, in which the prefrontal cortex has been implicated. The argument we make is that, unlike certain other thalamic nuclei (specifically, the first order nuclei), MD is not chiefly involved in relaying peripheral system information to cortex, but rather plays an important role in supporting the optimal performance of the interconnected cortex during specific cognitive functions, via the rapid transmission of relevant processed cortical messages to other dispersed cortical areas.

## The mediodorsal thalamus functioning in cognitive processes

Emerging perspectives on the thalamus and its functions have highlighted its role in modulating cortico-cortical information transfer, and suggest that higher-order thalamic nuclei (such as the MD and pulvinar) may be instrumental in relaying copies of efferent motor commands from one cortical region to another, via their respective transthalamic routes (Sherman, 2007). For a recent review of the role of the pulvinar in supporting visual attention processes across the cortex, see Halassa and Kastner (2017). One major source of difference between the influence of the MD on the cortex, compared with that of the pulvinar, is the distribution of thalamocortical projections from these two thalamic structures. As indicated above, the different subdivisions of the MD send axons to several areas within the frontal lobes and insular cortex. In contrast, the projections of the pulvinar are more widespread, targeting visual, parietal, and temporal lobes, in addition to the frontal and insular cortex (Romanski et al., 1997; Rockland et al., 1999; Kaas and Lyon, 2007; Halassa and Kastner, 2017). Thus, whilst we cannot draw strong conclusions from the thalamocortical connectivity alone, these observations provide a broad indication of the differing roles that the MD and pulvinar may play.

Here we will consider some of the evidence from the visual and sensorimotor systems, on which these proposals are based, and then extend these ideas to consider how the MD may also support cognitive operations attributed to the PFC.

Firstly though, it should be noted that even the sensory nuclei of the thalamus can in some sense be viewed as carrying an efference copy motor signal to the cortex, if we consider that many sensory structures also have a direct output to midbrain, brainstem, or spinal cord motor pathways. Interestingly, these efference copies have been identified across the animal kingdom (Crapse and Sommer, 2008) and may form a way of combining perception and action together via the thalamus in mammals (Guillery, 2005; Sommer and Wurtz, 2008). On the other hand, in the specific case of efference copies sent from cortical layer 5, the driver signal relevant to the MDmc and MDpc/MDcd, the thalamic nuclei that contain higher order relay cells that receive these collateralized copies of already processed cortical messages are defined as being higher-order by the origin of the cortical driver signal and the termination site of the axon, which as mentioned above is typically conceived of as a branch of a corticofugal axon that innervates a lower motor center in the midbrain (e.g., superior colliculus), brainstem or the spinal cord (Guillery et al., 1998; Sherman and Guillery, 1998). Thus, these higher order relay cells receive a copy of the processed efferent motor commands, which in turn they pass on to other areas of the cortex, perhaps to help prepare for a predicted upcoming motor response.

In the visual system, this information might be used to inform areas throughout the cortex about impending eye movements, in a process referred to as visual remapping. This involves the modulation of the receptive field properties of certain individual neurons immediately prior to a saccade, in order to align the internal representation of space with the new incoming retinal information. Remapping is thought to support spatial constancy and, though first identified in the parietal cortex, has now been observed in several different visual areas (Duhamel et al., 1992). The underlying mechanisms supporting this process continue to be investigated (Sommer and Wurtz, 2006; Neupane et al., 2016a,b; Rao et al., 2016).

Intriguingly, there is one particular, well-established computational framework, which closely resembles the actual transthalamic circuitry described above. Wolpert and Ghahramani (2000), describing an optimal sensorimotor control system more generally, propose the existence of a “Forward Model” in the central nervous system, which takes motor commands (using efference copies) as its input and calculates the sensory signals that are likely to be produced as a result of any impending movements (termed the “reafference”); this output can then be relayed to other parts of the brain for use in several different computations, as discussed below. The forward model also works in concert with an “inverse model,” which performs the translation in the opposite direction; that is, the inverse model starts with a desired outcome and selects the appropriate movements required to achieve this (Stein, 2009), and is thus based on prior experience.

Whilst the forward model, unlike the inverse model, is not strictly necessary for a sensorimotor system to function, it does make several important contributions to the efficiency with which movements can be controlled and carried out: firstly, the forward model means that the predicted future state of the system is immediately accessible and can guide fast, online updating of motor commands, whereas waiting for direct sensory feedback introduces a time delay of several tens to hundreds of milliseconds (Miall and Wolpert, 1996). Moreover, the forward model allows those portions of the sensory signal that reflect actual changes in the world to be distinguished from those that result from self-generated actions, supporting accurate perception. Finally, it may allow for the attribution of agency to sensory events, since a common assumption in theoretical models is that, depending on the extent to which the actual reafference signal matches the output of the forward model, a movement can be attributed to the self (Wolpert and Flanagan, 2009), whereas if these two signals are discrepant it must reflect the action of some external force or agent. By the same token, damage to the forward model, or rather to the anatomical structures underlying it, is likely to have a significant functional impact across several domains, including movement, perception, and the attribution of agency.

Just as in the earlier work on visual remapping, it has previously been suggested that this framework is implemented through direct cortico-cortical communication. Frith (2005), for example, suggested in his model of schizophrenia that forward modeling involves communication between areas of the brain responsible for motor outputs and areas responsible for sensation, with the one sending a copy of efferent motor commands directly to the other. Alternatively, others have more recently proposed the transthalamic route, via the higher order nuclei, as a potential neural correlate of the forward model (Sherman and Guillery, 2006, 2013). Intriguingly, support for this suggestion (albeit indirectly) comes from the observation that cortico-thalamic and thalamo-cortical axons have a larger diameter and greater myelination than direct cortico-cortical projections (Salami et al., 2003) making them best placed to facilitate the fast transfer of an efference copy well ahead of any sensory reafference, as is required by the forward model or visual remapping; or, as indicated below, in various aspects of cognitive control.

In relation to aspects of cognitive control, neuropsychologists and computational modelers have proposed that different parts of the dorsal thalamus are important for coordination of oscillatory activity across their respective neural networks for successful memory retrieval to occur (Staudigl et al., 2012; Ketz et al., 2015). Oscillatory patterns have been investigated for several decades now and it is known that synaptic plasticity is regulated by synchronous and non-synchronous neural oscillatory activity within and between brain networks (Buzsáki and Draguhn, 2004; Lopes da Silva, 2013). Further, in humans, better memory performance has been observed with decreases in beta frequency (Hanslmayr et al., 2012, 2014; Meconi et al., 2016) and alpha frequency (Hanslmayr et al., 2012) neural oscillations. Several studies investigating cognitive functioning have noted that improved performance correlates with oscillatory changes in the left hemisphere of the dorsolateral PFC (Staudigl et al., 2012; Meconi et al., 2016; Guimond et al., 2017). Interestingly, researchers have also noted that patients with schizophrenia, who frequently present with impaired working memory, do not display this reduction in beta frequency neural oscillatory power, which the researchers propose causes the patients' deficits in memory encoding (Haenschel et al., 2009; Hanslmayr et al., 2012, 2014; Meconi et al., 2016; Guimond et al., 2017).

Thus, given all of these above proposed models of brain functioning, how might the MD be involved in supporting the PFC in higher cognitive functions? One possibility related to the influence of the MD in cognitive control, working in partnership with the PFC, is that it may support optimal task-relevant oscillatory regulation of cortical neurons, and it may do this rapidly via the transthalamic route of information transfer, proposed by Sherman and Guillery (2006). Further research will need to determine if this is indeed so. Nevertheless, we know based on the neuroanatomy that the different subdivisions of the MD, receive many excitatory and inhibitory inputs, and these inputs may help to regulate the coordination of these cortical oscillations, with the MD (as other higher order nuclei are proposed to do) operating as a “gate” (Sherman and Guillery, 2006, 2013) through which information may or may not pass, depending on the modulatory balance between these interconnections at the critical time (Llinás and Paré, 1991; Sherman, 2016). This proposal also implies that the influence of the MD on the cortex will be rapid (as is shown in the causal behavioral evidence outlined below), and that it has an ongoing, interactive influence, again as supported by its unique neuroanatomical interconnections.

One inhibitory pathway that is of particular interest in its ability to control the responsivity of thalamic relay cells is the GABAergic pathway linking the thalamus and the reticular thalamic nucleus (Halassa and Acsády, 2016). All cortical inputs and outputs between the thalamus pass through the reticular thalamic nucleus: notably though, whilst the layer 6 corticothalamic modulatory inputs send their collateral branch to terminate in the reticular thalamic nucleus, the layer 5 corticothalamic driver inputs simply pass through with their collateral branch terminating in the specific thalamic nucleus relevant to that driver signal (Jones, 2012). One further important point of note is that the rodent and primate thalamus are distinctly different, with very few GABAergic interneurons present in the rodent thalamus apart from in the lateral geniculate nucleus (Arcelli et al., 1997). Therefore the GABAergic inhibitory mechanisms exerted on the thalamus from the reticular thalamic nucleus will likely be very different in rodents to those in primates, who have the GABAergic inhibitory mechanisms exerted from the reticular thalamic nucleus, as well as from local thalamic GABAergic interneurons.

Now, behavioral evidence as detailed in the next sections is beginning to show that the different subdivisions of the MD have different, interdependent roles in cognitive functions, with the apparent overall common function being that they are involved in rapidly updating ongoing cognitive processes governed by the frontal lobes. Specifically, the lateral MD has been implicated in supporting the frontal cortex to perform rapid updating of saccadic eye movements, whilst the MDmc is thought to be involved in the rapid updating of information that the frontal cortex needs for optimal new learning and adaptive decision-making to occur. However, precisely how the different subdivisions of the MD are interacting with the cortex to accomplish this dynamic, online updating is still unknown and continues to be investigated.

### Evidence of upcoming motor commands—primates

By incorporating the thalamus in the motor-control pathway, the entire interconnected cortical network can anticipate a movement and make necessary compensatory measures for its occurrence. If the thalamic relay is no longer present or disrupted then this anticipatory signal is not transmitted or is transmitted in a non-viable manner that may make it impossible, or very difficult for the cortical network to predict what to do next. The consequence of this is that suboptimal behavioral and cognitive performance will occur. Models involving efference copies for the purpose of motor control are often only hypothetical, and as such fail to identify the specific anatomical structures that may carry out each role (Guillery and Sherman, 2011). Nonetheless, there is now an emerging body of evidence suggesting that the efference copy mechanism *does* occur in practice, and that the thalamus is likely employed to complete this function. For example, Sommer and Wurtz (2006) trained primates in a visual probe task that involved making a saccade to a target in the periphery of a central fixation point. They observed that prior to the execution of the saccade, neurons in the frontal eye fields (FEF) shifted their receptive fields to the saccade destination. It was hypothesized that the lateral MD nucleus propagates corollary discharges from the superior colliculus forward to FEF, enabling them to shift their receptive fields in anticipation of the saccade, a form of motor driver originating in the superior colliculus that is transmitted through the lateral MD onto cortical regions (Wurtz et al., 2005). Through this, smooth spatial visual processing can occur. Interestingly, this evidence suggests that the lateral MD may perhaps be a first order relay as its driver signal originates in a brainstem site (Wurtz et al., 2005) rather than in layer 5 of the cortex. They confirmed that the relay of signals from the lateral MD nucleus to the FEF causes the receptive fields to shift, as monkeys could no longer shift their receptive fields after temporarily inactivating the lateral MD using muscimol, a GABA-agonist. Disrupting the corollary discharge process in this way meant that the FEF receptive fields could not shift in preparation of the saccade, potentially due to the interruption of transthalamic forward signaling, a mechanism that may have ramifications for many neuropsychiatric disorders (Sommer and Wurtz, 2008). Sommer and colleagues have continued to show how this pathway is crucial for signaling where the monkey's eyes are pointing, especially during sequences of saccades and when stimuli are changing in the visual receptive field (Crapse and Sommer, 2012; Mitchell et al., 2014).

### Evidence of upcoming response changes after MDmc damage—primates

In cognitive and behavioral studies in monkeys, subtle but key deficits emerge after selective damage to the magnocellular subdivision of the mediodorsal thalamus (MDmc). Work in the Mitchell lab over the past decade has established the importance of an intact MDmc in cognitive tasks that require the monkeys to rapidly process trial relevant task information when involved in new learning or adaptive decision-making (Mitchell et al., 2007a,b, 2008, 2014; Browning et al., 2015; Chakraborty et al., 2016). In contrast, an intact MDmc was not required when the monkeys needed to retrieve pre-operatively acquired information, or when they needed to implement a different response strategy for reward, or for maintenance of working memory, attention or motivation to complete the task *per se* (Mitchell et al., 2007a; Mitchell and Gaffan, 2008; see also Table 1).

**Table 1:** Cognitive and behavioral effects after selective lesions to the mediodorsal thalamus in non-human primates (rhesus macaque monkeys).

**Study**	**Lesion to**	**Task**	**Effect**
Isseroff et al., 1982	Bilateral MD – ablations	Spatial delayed alternation	Impaired
		Spatial delayed response	Impaired
Aggleton and Mishkin, 1983	Bilateral MD – ablations	Object recognition memory	Impaired
		Object-reward associations	Impaired
Zola-Morgan and Squire, 1985	Bilateral MD – ablations	Delayed non-match to sample	Impaired
		Pattern discriminations	Not Impaired
Parker et al., 1997	Bilateral MDmc – ablations	Object recognition memory	Impaired, if large numbers of objects used
Gaffan and Parker, 2000	Bilateral MDmc – ablations	Learnt 20 novel object-in-place scene discriminations within a session	Impaired
Gaffan and Parker, 2000	Bilateral MDmc – ablations	Learnt object-reward associations within a session	Impaired
Mitchell et al., 2007a	Bilateral MDmc – neurotoxins	Learnt 20 novel object-in-place scene discriminations within a session	Impaired–increased switching not perseverative responding
Mitchell et al., 2007a	Bilateral MDmc – neurotoxins	Implementation of preoperatively acquired strategy	Not Impaired
Mitchell et al., 2007b	Bilateral MDmc – neurotoxins	Learnt 60 object-reward associations across sessions to criterion	Not Impaired
Mitchell et al., 2007b	Bilateral MDmc – neurotoxins	Food devaluation	Impaired
Mitchell and Gaffan, 2008	Bilateral MDmc – neurotoxins	Retention of 300 preoperative acquired object-in-place scene discriminations	Not Impaired
Mitchell and Gaffan, 2008	Bilateral MDmc – neurotoxins	Postoperative learning of 100 novel object-in-place scene discriminations across sessions	Impaired
Mitchell et al., 2008	Bilateral MDmc – neurotoxins + fornix transection	Retention of 300 preoperative acquired object-in-place scene discriminations	Impaired, combined damage to two interdependent neural circuits caused retention deficits
Mitchell et al., 2008	Bilateral MDmc – neurotoxins + fornix transection	Postoperative learning of 100 novel object-in-place scene discriminations across sessions	Impaired, greater deficit compared to bilateral MDmc lesions alone–Mitchell and Gaffan, 2008
Izquierdo and Murray, 2010	Unilateral MDmc – neurotoxic + contralateral amygdala + orbitofrontal cortex	Food devaluation	Impaired
Browning et al., 2015	Unilateral MDmc – neurotoxins	Learning 20 novel object-in-place scene discriminations within a session	Impaired
Browning et al., 2015	Unilateral ventrolateral PFC and orbitofrontal cortex ablation	Learning 20 novel object-in-place scene discriminations within a session	Not impaired
Browning et al., 2015	Contralateral ventrolateral PFC and orbitofrontal cortex X MDmc – neurotoxins	Learning 20 novel object-in-place scene discriminations within a session	Impaired
Browning et al., 2015	Contralateral ventrolateral PFC and orbitofrontal cortex X MDmc – neurotoxins	Food devaluation	Impaired
Browning et al., 2015	Ipsilateral ventrolateral PFC and orbitofrontal cortex + MDmc – neurotoxins	Learning 20 novel object-in-place scene discriminations within a session	Not impaired
Browning et al., 2015	Ipsilateral ventrolateral PFC and orbitofrontal cortex + MDmc – neurotoxins	Food devaluation	Impaired
Chakraborty et al., 2016	Bilateral MDmc – neurotoxins	Learning novel 3-choice probabilistic object-reward associations within a session and reversals	Impaired–increased switching. Disrupted rapid updating of next choice response based on recent choice history

In relating these deficits in cognitive performance after MDmc damage to a mode of transthalamic information transfer (or aspects of cognitive control), our recent causal evidence is able to help, whereby monkeys with damage to the MDmc demonstrated deficits when required to incorporate their own recent choice history (i.e. what choice they made on trials n-1, n-2, and n-3 back in time) to guide their own upcoming optimal choice response during adaptive decision making (Chakraborty et al., 2016). That this is happening within this short epoch of time, suggests the importance of the MDmc for supporting the rapid updating of PFC representations in line with what has just occurred in the task. With the MDmc transthalamic route impaired, the monkeys increased their switching behavior, sampling all three objects (but not randomly), implementing an unsuccessful exploratory style of responding (win-shift) after the reversal of object-reward contingencies that created a need to adapt behavior within the testing session (Chakraborty et al., 2016).

This behavior suggests that information transferred via the MDmc transthalamic route supports fast (vs. slow) updating of PFC representations related to the ongoing, fluid aspects of the task relevant information. Further, in relation to the (Chakraborty et al., 2016) study, the multiple regression analyses of the data relevant for the objects, rewards and choices indicated that the monkeys with damage to the MDmc had difficulties updating their recent choice history, which in line with the current hypothesis suggests that an efference copy signal from layer 5 of cortex, that is branching to the MDmc, is transmitting a processed motor command signal to foretell other cortical areas about the predicted upcoming response. Thus, based on this behavioral evidence, we speculate that this particular transthalamic route via the MDmc is important for the cortical updating of task relevant information related to upcoming choices. This hypothesis about the MDmc being important for updating rather than the active maintenance of the old information *per se* (Mitchell, 2015), is also supported by the lack of impairments when monkeys with MDmc damage showed good performance when only required to retrieve pre-operatively learned task relevant information across different cognitive tasks (Mitchell et al., 2007a; Mitchell and Gaffan, 2008).

### Neural oscillations in rodents

Evidence from rodents also indicates that the MD and cortex work together to support successful cognitive processing. However, most of the rodent literature has not yet differentiated the subdivisions of the MD in their studies (although see Mitchell and Dalrymple-Alford, 2005, 2006). Successful recency recognition memory in rats, for example, is critically dependent on the integrity of MD-mPFC circuitry (Cross et al., 2013), highlighting how the interplay of communication within and between MD-mPFC networks is crucial for cognition.

Other rodent studies have attempted to examine precisely how changes to MD activity could disrupt functional communication within the MD-PFC circuit, resulting in deficits in cognitive processing. A study by Parnaudeau et al. (2013), which combined pharmacogenetics with *in vivo* recording in mice, provides evidence for a functional dissociation of thalamo-prefrontal substrates for working memory maintenance and retrieval. They showed that decreasing the activity of MD neurons was sufficient to induce selective impairments in two PFC-dependent cognitive tasks.

Interestingly, simultaneous electrophysiological recording between MD and PFC during the delayed nonmatching-to-sample task revealed that the spiking of individual MD neurons showed synchrony with mPFC local field potentials according to choice accuracy during learning phase. After successful acquisition, beta frequency synchrony (13–30 Hz) was specifically enhanced in the working memory-requiring choice phase of the task. Furthermore, reducing MD activity delayed the strengthening of synchrony between MD and mPFC, particularly within the beta frequency range, suggesting a functional dissociation of thalamo-frontal substrates for spatial working memory (Parnaudeau et al., 2013).

Another example of MD's role in supporting cortico-cortical communication is the work first conducted by Eichenbaum et al. (1980) and then developed later by Courtiol and colleagues (Courtiol and Wilson, 2014, 2016; Wilson et al., 2014). Eichenbaum et al. (1980) showed that rats with lesions of the MD exhibit deficits in difficult odor driven discrimination tasks. For instance, rats with MD lesions need more trials to reach the discrimination criterion within the session when the task difficulty is increased (Eichenbaum et al., 1980). Functionally, the study by Courtiol and Wilson (Courtiol and Wilson, 2014) showed that in response to either monomolecular, mixture or biological odorants in anesthetized animals, both the MD and its primary olfactory afferent, the piriform cortex, express beta oscillations; these oscillations were coherent between the two structures, and importantly, some MD units fired in phase with piriform cortex beta (Courtiol and Wilson, 2014). With respect to discrimination, both single units and local field potentials, recorded in MD, piriform cortex, and orbitofrontal cortex in rats performing a two-alternative odor discrimination task, revealed that subsets of MD units display odorant selectivity during sampling, as well as encoding of spatio-motor aspects of the task (Courtiol and Wilson, 2016). Furthermore, the olfactory transthalamic network rapidly switches functional connectivity between MD and cortical areas depending on current task demands, with, for example, MD-piriform cortex coupling enhanced during odor sampling and MD-OFC coupling enhanced during the decision/goal approach compared with baseline and presampling (anticipation) (Courtiol and Wilson, 2016). Intriguingly, patients diagnosed with various neuropsychiatric disorders also often present with odor detection problems, as discussed later under section Neurodevelopmental Disorders.

More recently, researchers using optogenetic tools coupled with *in vivo* recordings in mice have provided the first indication of how the lateral MD may sustain prefrontal representations of abstract rules without relaying categorical information. Indeed, they have shown for the first time in rodents that the lateral MD thalamic circuit operates as a cortical amplifier for local cortical connectivity sustaining attentional control (Schmitt et al., 2017). This causal evidence links well with our proposal about the importance of the ongoing, dynamic partnership between the MD and the cortex during cognitive processes to support optimal performance. Further, their evidence demonstrates that enhancing lateral MD excitability seems to increase PFC rule information content (as measured with behavioral performance differences in a two-alternative forced choice cognitive task) by improving tuning of individual cortical neurons, and by recruiting previously untuned ones (Schmitt et al., 2017).

Clearly, rodent studies have been informative in the debate about the functions of the primate MD. However, there are inherent difficulties in using rodents as a model of primate cognitive function. For example, the morphology of the thalamus and the complexity of the laminar structure of the cortex are different across these two mammalian species. Thus, the key question that is raised here is whether the cortico-thalamo-cortical related cognitive functions are distributed in a similar fashion between primates and rats. Interestingly, based on anatomical and behavioral evidence, Kolb (Kolb, 1990; Uylings et al., 2003) suggested that the medial part of the rat PFC might subserve some of the cognitive functions localized to the dorsolateral PFC in primates. Nevertheless, the rodent cortex lacks the granular and agranular complexity of the primate, and especially the human brain. Therefore, we think that before extrapolating rodent data to primates, we should consider the fact that the same cognitive operations and analogous neural substrates could mediate different behaviors across species. Thus, we strongly recommend identifying common fundamental operations under species-appropriate conditions rather than studying apparently similar behaviors.

### Cortical changes after early MD lesions in rodents

If higher cognitive abilities typically conceived of as being governed by the PFC are regulated in part by interactions with the MD then a potential avenue for understanding MD-PFC interactions is to investigate how this communication is established during neurodevelopment. To our knowledge, this work has so far only been performed in rodent models. In these previous studies, it has been established that the cognitive and behavioral effects of early MD lesion are critically dependent on the timing of the lesion. The early postnatal period (from postnatal days P1 to P10) in the rat brain is characterized by dramatic changes contributing to synaptic formation, growth, regression, and stabilization of connections (Cotman and Nieto-Sampedro, 1984). In the early postnatal weeks, the PFC matures both morphologically and functionally. Thus, at postnatal day P7, all cytoarchitectural layers of the PFC become distinguishable, but are still very immature (van Eden and Uylings, 1985). Data from previous studies on brain maturation in rodents suggests that manipulations that occur to the neuronal structures prior to day P7 produce permanent changes in the brain that persist into adulthood. In contrast, manipulations occurring on or after day P7 do not cause similar long-term changes in the brain, suggesting that these later changes have little or no effect on the development of the cortex (Gabbott et al., 2003) or that a form of neuroplasticity or recovery of function has intervened given that the basic laminar structure has been established. In addition, previous studies have demonstrated that when performed either directly after birth on postnatal day 0 (P0) or at day P7, MD manipulations have no effects either on behavior or on the morphology of the PFC (van Eden et al., 1994; Lipska et al., 2003), suggesting that if performed at P0 then neurons are able to migrate via a different route to the cortex. Therefore, Ouhaz et al. (2015, 2017) devised an animal model to test the longer-term effects of early developmental insult to MD on the onset of changes to behavior and in some domains of cognition. MD lesions were conducted on day P4, which coincides with the time window when afferent and efferent connections are forming between the MD and PFC (van Eden, 1986).

Adult rats that had previously sustained neonatal MD lesions performed at day P4 showed selective deficits in a battery of cognitive and behavioral tasks (Ouhaz et al., 2015). For example, they were impaired in the ability to switch from an innately preferred strategy (nonmatching/alternation) to a new strategy (matching), as reflected by a specific increase in perseverative errors (Ouhaz et al., 2015). The early MD lesions also disrupted acquisition during the passive avoidance test indicating that an intact MD is important during the initial stages of learning in this task (Ouhaz et al., 2015). Further, another group of adult rats that had sustained their MD lesion at day P4 showed reduced recognition memory (Ouhaz et al., 2017). However, there is little evidence for a specific loss of recognition memory in adult rats with MD lesions; instead, more consistent deficits have been found for object recency, and this may reflect the close functional link between the MD and the PFC in the adult brain (Hunt and Aggleton, 1998; Mitchell and Dalrymple-Alford, 2005; Barker et al., 2007). In addition, the MD lesions made at postnatal day P4 resulted in increased anxiety-like behavior in adulthood as seen in the elevated plus maze test and by assessing thigmotaxic behavior during the exploration of open field apparatus (Ouhaz et al., 2017). Similar results have been reported both in a previous study using the same procedure (Ouhaz et al., 2015), in humans when anxiety levels are manipulated (Bishop et al., 2004), and in mice with MD lesions performed in adulthood (Chauveau et al., 2005), thus suggesting that the increase in anxiety-like behavior is not necessarily developmentally driven (Ouhaz et al., 2015, 2017).

Finally, early MD lesioned animals show deficits in social behaviors, whereby they spent less time engaging in social interactions during adulthood (Ouhaz et al., 2015). Interestingly, lesions to the PFC at differing stages of development in rodents have reported contrasting effects. Dopaminergic lesions of the medial PFC during adolescence caused an extensive impairment in socially interactive behavior, while medial PFC lesioned adult rats spent significantly longer periods of time in social interactions (Maaswinkel et al., 1996; Shah and Treit, 2003), suggesting an anxiolytic-like effect on behavior following damage to the medial PFC in adulthood. Our data suggest that the behavioral effects linked to early onset damage during brain maturation in the MD is comparable to damage in the medial PFC sustained during adolescence (Shah and Treit, 2003) in leading to impaired social interactions during adulthood.

The principal advantage of early lesion models is their ability to capture a number of key features attributed to some psychoaffective disorders such as schizophrenia, namely its developmental pathogenesis, its cognitive deficits, and the neuroanatomy beyond its emergence. For example, Goldman (1971) first showed that perinatal ablations of the PFC in primates did not impair performance on a delayed response task until after adolescence. However, animal models inducing a lesion early on in the brain's development lack construct validity, as the schizophrenic brain does not manifest a “lesion” analogous to these models. Nevertheless, the neurodevelopmental damage approach has generated animal models with great heuristic value, in particular for discovering neural and behavioral consequences of the initial injury distal to it both anatomically and in terms of developmental timeline (Tseng et al., 2009; Ouhaz et al., 2015).

Finally, as the PFC has an extended maturation period, a sustained window of plasticity may extend the developmental time window, during which an MD lesion may affect the circuitry (Uhlhaas et al., 2013). The research investigating the effects of early MD lesions has firmly challenged the dogma of the MD's role as a passive relay, and has instead suggested that the MD has a core function in establishing and updating newly learned information for use by the PFC. We therefore hypothesize that MD efferent activity is important for the regulation of PFC development earlier than previous data has indicated.

## Neurodevelopmental disorders

As an understanding of the functions of the higher-order thalamic nuclei has begun to emerge, researchers have also become interested in the deficits that may result when these functions are disrupted, and thus the role that thalamic dysfunction may play in a number of neuropsychiatric disorders. Much of this research has focussed on schizophrenia; however, more recently there have also been attempts to apply our knowledge of the thalamus to inform research on other disorders, such as for example, autism. We outline some of this work below.

### Schizophrenia

#### Overview of the disease and the rationale for considering the thalamus

Schizophrenia is a severe neuropsychiatric disorder that contributes significantly to the burden on public health worldwide. Whilst a diagnosis of schizophrenia requires the presence of positive symptoms, namely hallucinations or delusions, typically also accompanied by negative symptoms such as apathy or withdrawal (American Psychiatric Association, 2013), cognitive symptoms are also now increasingly important to our understanding of the disease and seem to constitute a core feature (Mesholam-Gately et al., 2009). Further, they deserve particular emphasis since they may predict poor functional outcome more strongly than either positive or negative symptoms alone (Green, 1996; Green et al., 2000; Tabarés-Seisdedos et al., 2008), and remain refractory to current treatments (Hagan and Jones, 2005; Goldberg et al., 2007).

Frith and colleagues (Frith et al., 2000; Frith, 2005) have proposed a model of schizophrenia to account for a specific subset of the positive symptoms, namely delusions of control, in which the patient feels that an external force or agent is controlling their actions. Because the attribution of agency to sensorimotor events is believed to rely upon the forward model (see above section Evidence of Upcoming Motor Commands—Primates), it was proposed that these symptoms may reflect impaired forward modeling. Later work has suggested that this same idea might also be extended to explain other symptoms such as auditory hallucinations as well (Feinberg, 2011).

Interestingly, dysfunction of corollary discharge circuitry has been identified in patients diagnosed with schizophrenia (Feinberg, 1978; Feinberg and Guazzelli, 1999), with the underlying mechanisms still being investigated, including assessments of eye movements (Ford et al., 2001; Ford and Mathalon, 2004; Nawani et al., 2014; Pack, 2014; Richard et al., 2014). Nevertheless, as was noted earlier, there is a clear similarity between the proposed (computational) forward models and the actual anatomy of the first order and higher order nuclei of the thalamus, with at least some of the driver messages that these nuclei receive conveying efference copies/corollary discharges. Therefore, it has subsequently been suggested by others that dysfunction of the thalamus could provide the basis for forward modeling deficits (Sherman and Guillery, 2006; Sherman, 2016) and, by extension, contribute to some of the symptoms of schizophrenia (Vukadinovic, 2011; Sherman and Guillery, 2013). In support of this proposal, substantial evidence is accumulating that the thalamus is affected early on in the development of schizophrenia, and moreover that some of these changes may also be relevant for explaining the cognitive symptoms of the disease, which are as yet relatively poorly understood.

#### Current models to explain thalamic changes in schizophrenia

Whilst there are a wide variety of brain alterations that have been associated with schizophrenia, many neural models were built upon Kraepelin's notion that the clinical features of the illness are explained best by pathology of the cortex, primarily the PFC (Kraepelin, 1919). However, thalamic pathophysiology appears to be a core feature that is present from before the onset of clinically detectable symptoms, and this may drive the alterations observed in the other parts of the brain. This suggestion comes principally from four sources. First, the functional neuroanatomy of the thalamus (as described in section Volumetric Changes and White Matter Differences), with several groups of nuclei establishing extensive reciprocal connections with cortical regions, subcortical regions, and the cerebellum, lends itself to sophisticated neural models of neuropsychiatric illnesses such as schizophrenia (Andreasen, 1997; Jones, 1997; Scheibel, 1997). Clinical symptoms of schizophrenia such as perceptual disturbances, motor anomalies, cognitive deficits, and vegetative disturbances point toward functions subserved by the human thalamus, including sensory relay, motor relay, maintenance and regulation of consciousness, attention and memory (Schmahmann, 2003). Second, some studies have linked the psychotic features of schizophrenia to a breakdown of the sensory filter or gating role of the thalamus (both total and subnuclear such as the MD, centromedian thalamic nuclei, pulvinar and anterior thalamic nuclei; Carlsson and Carlsson, 1990; Jones, 1997). Moreover, several studies have associated the emergence of negative symptoms, attention impairments, and executive dysfunction to thalamic changes (Salgado-Pineda et al., 2003; Preuss et al., 2005; Crespo-Facorro et al., 2007). Third, the well-known pathology of the PFC in schizophrenia has led some to implicate the closely connected MD (Goldman-Rakic and Porrino, 1985; Popken et al., 2000; Fuster, 2015), or it could point to a pathology of primarily those layers (layers 3 and 4) in the PFC that are receiving projections from the MD (Lewis et al., 2001). Such a model implies selective thalamic changes in the MD, including structural and functional abnormalities. Finally, some have argued for an abnormal role of the thalamus in a distributed network of cortical-subcortical neural circuits. Disruptions were proposed to occur in the cortical-cerebellar-thalamic-cortical circuitry leading to cognitive deficits in sensory perception, memory encoding and retrieval, and the prioritization of daily experience and information (Andreasen et al., 1996, 1998). This model implies that thalamic abnormalities are more likely to occur in the presence of other structural or functional changes within an abnormal distributed neural network and not in isolation.

#### Volumetric changes and white matter differences

There is substantial evidence that the volume of the thalamus is decreased in patients with schizophrenia (for reviews, see Alelú-Paz and Giménez-Amaya, 2008; Byne et al., 2009). Post-mortem studies indicate that the gray matter volume of the dorsal thalamus is reduced; specifically, the MD and pulvinar higher order thalamic nuclei, and the anterior and midline thalamic nuclei (Young et al., 2000; Danos et al., 2003, 2005). Results of structural magnetic resonance imaging (MRI) studies are more mixed, but this may in part be a result either of low sample sizes or because relatively poor spatial resolution in early studies precluded the assessment of individual thalamic nuclei (Alelú-Paz and Giménez-Amaya, 2008). With the increased resolution of MRI now able to resolve individual thalamic nuclei, as well as the use of larger samples (e.g., van Erp et al., 2016) and more sophisticated statistical analyses (e.g., Pergola et al., 2017), recent MRI studies have more consistently provided support for the idea that overall thalamic volume *in vivo* is significantly reduced in schizophrenia (with only decreased hippocampal volume and increased lateral ventricular volume showing larger effect sizes (Byne et al., 2009)), and that this is driven particularly by effects in the higher order thalamic nuclei. This change is present from early on, before the manifestation of clinical positive and negative symptoms of the disease, and has also been detected (with a smaller effect size) in genetically at-risk populations, such as first-degree relatives (Pergola et al., 2017).

In addition, the white matter tracts connecting MD to the PFC have been shown to have greater fractional anisotropy (a proxy for the relative diffusion of signals being transmitted in the white matter pathways of the brain) in schizophrenic patients than in healthy controls, suggesting a dysfunctional reciprocal connectivity between this major thalamic relay nucleus and its target sites in the cortex (Kito et al., 2009). Andreasen et al. (1994) reported evidence for co-occurring pathology in the thalamus and the white matter tracts leading to the dorsolateral PFC; this was replicated later with diffusion tensor imaging of the thalamic radiations to the cortex (Kito et al., 2009).

In patients diagnosed with schizophrenia, postmortem cytopathological descriptions of the MD are conflicting, but most tend to show smaller neuron numbers without gliosis or any other hallmark signs of neurodegeneration (Heckers, 1997). This argues for a process that is not degenerative, but perhaps developmental in origin. The first high-quality stereotactic cytopathological studies of the thalamus reported lower numbers of both neurons and glial cells in the MD, independent of medication effects (Pakkenberg, 1990, 1992). These studies and an interesting report from Young and colleagues (Young et al., 2000) found profound reductions in the number of neurons in the MD when comparing people with schizophrenia to controls. The findings were even more striking because there was virtually no overlap between groups.

#### Functional imaging results

Studies utilizing functional imaging indicate that the overall levels of activity in the thalamus are abnormal (although whether this is hypo- or hyper-activity appears to depend on the specific task used; Byne et al., 2009); and that there is a functional disconnection between MD and the PFC (Schlösser et al., 2003; Giraldo-Chica and Woodward, 2017). A concomitant increase in the resting-state functional connectivity with sensory and motor cortices has also been reported, which strongly negatively correlated with the degree of MD-PFC disconnection (Anticevic et al., 2014; Woodward and Heckers, 2016), suggesting that both effects are possibly being driven by the same mechanism. This dual effect was present in a sample of young people genetically at-risk for schizophrenia, and was strongest amongst those who, at a two-year follow up, had gone on to develop psychosis (Anticevic et al., 2015). Furthermore, activation in distributed thalamocortical networks has been observed in actively hallucinating patients (Silbersweig et al., 1995).

#### Changes in oscillatory activity

In schizophrenia, both the amplitude and coherence of beta- and gamma-band oscillations have been shown to be decreased during the execution of cognitive tasks, and the gamma decrease in particular has been correlated with impaired performance in a range of domains including working memory (Haenschel et al., 2009), executive control (Minzenberg et al., 2010), and perception (Ford et al., 2008; Hirano et al., 2008). Whilst fewer studies have examined the functional effects of beta-band reductions in schizophrenia, it has been suggested that they are associated with impaired perceptual integration (Uhlhaas et al., 2006). An intriguing result also comes from Ford and colleagues (Ford et al., 2007), who reported that the increase in beta-band synchrony usually observed in healthy participants immediately prior to self-generated speech (as opposed to when listening to a recording of themselves speak) is reduced in schizophrenia patients, especially those with active auditory hallucinations. This provides another link to the proposed forward modeling deficits, in addition to suggesting that the ongoing, interactive updating necessitates the rapid interactions between the cortex and the thalamus via the transthalamic routes.

### Understanding the cognitive symptoms

The forward modeling framework of Frith (2005) provides a persuasive account of how the thalamic alterations summarized in the preceding sections can be linked to hallucinations and delusions of control. Elaborating on this idea, we believe that dysfunction of the higher-order nuclei of the thalamus may also contribute to the cognitive deficits seen in schizophrenia as well. In particular, the proposed role of the thalamus in cognitive control is thought to be implemented through the regulation of cortical synchrony, allowing selective management of functional connectivity and thus flow of information through the cortex. For example, Saalmann and colleagues have shown, using simultaneous multi-unit recording in monkeys, that the pulvinar interacts with cortical areas V4 and TEO by regulating alpha-band synchrony in line with visual attention demands (Saalmann et al., 2012). Additionally, Wilke and colleagues have demonstrated, using temporary unilateral inactivation of the dorsal pulvinar in monkeys, that this higher-order nucleus contributes to updating internal assessments of the desirability of competing saccade targets in the contralesional and the ipsilesional hemifield, and so supports oculomotor decision making (Wilke et al., 2013).

We should highlight that despite the phylogenetic and developmental commonalities between the MD and pulvinar (Walker, 1966), their specific neuronal interconnectivity and structural organization provides clues to their differences (Jones and Hendry, 1989). For example, concerning PFC efferents, tract tracing studies have revealed that PFC cortico-pulvinar projections originate exclusively from layer 6 (Romanski et al., 1997). For the pulvinar (lateral posterior nucleus in cats and rodents), layer 5 cortical inputs are received from several visual cortical areas, namely 17, 18, and 19 (Abramson and Chalupa, 1985; Sherman and Guillery, 2002). In addition, the study of Jones and Hendry (1989) showed the existence of different populations of relay neurons in the monkey thalamus according to their immunoreactivity for parvalbumin and calbindin. Interestingly, the pulvinar showed homogeneously distributed and co-localized expression of parvalbumin and calbindin in the neuropile and in the somata. In contrast, in the MD, parvalbumin was expressed exclusively within the MDmc subdivision, whereas calbindin was expressed homogeneously across all subdivisions with a slight increase in the MDmc (Jones and Hendry, 1989). In light of this, we can conclude that MD and pulvinar, despite both being classified as higher order nuclei, are key nodes in independent neural networks and will thus provide different functional contributions to distinct cognitive and executive functions.

Nevertheless, the implication of these studies is that, if the functioning of the pulvinar or MD is impaired, as seems to be the case in schizophrenia, then there are likely to be deficits in the deployment of visual attention or higher order cognitive functions governed by the PFC, respectively. Possibly then, damage to the higher order thalamic nuclei and their interconnections with the cortex could be part of the underlying mechanism that directly causes many of the cognitive symptoms seen in schizophrenia. Alternatively, or perhaps even additionally, thalamic damage during development could cause cortical dysregulation and, in turn, cortical damage, with further deleterious effects on cognitive processing (as discussed in section Cortical Changes after Early MD Lesions in Rodents).

Clearly there is a great deal more research required in order to understand how animal studies such as these can translate to actual human patients. Nevertheless, this initial work at least provides a good indication that investigating the thalamus may prove enlightening for researchers studying and, ultimately, seeking to treat schizophrenia.

### Autism and other neuropsychiatric disorders

In previous decades, social factors or parenting styles were the dominant explanations used to account for the underlying deficits linked with autism. However, these explanations have fortunately also had to be substantially altered owing to the identification of genetic risk factors and neuronal changes, as well as the identified changes in sensory processing for people diagnosed with autism (Frith et al., 1991; Frith, 1993; Frith and Frith, 1999). Now, evidence is beginning to emerge that there is a neurobiological basis, potentially involving key changes in transthalamic routes (Chen et al., 2016; Woodward et al., 2017), although very few studies have yet provided specificity linked to the MD thalamus. Nevertheless, the key message that we propose is that perhaps disruptions occur early on during neurodevelopment to cortico-cortical information transfer, due to alterations in the connectivity between the thalamus and the cortex. Given this, significant changes may also occur in the transthalamic routes linking cortico-cortical communication and consequently may contribute to many of the cognitive disruptions evident in other neuropsychiatric disorders. For example, the MD is the key thalamic node in the olfactory system, although it is not a primary relay (see Figure 1) and amongst several neuropsychological conditions, including Alzheimer's disease, Parkinson's disease, schizophrenia, autism and depression, disrupted odor perception is a common complaint (Tham et al., 2009; Wilson et al., 2014). Further, thalamic stroke patients with damage to the MD, or patients with epilepsy that has its foci in the anteromedial temporal lobes (that project to the MDmc) have problems with odor detection (Tham et al., 2011; Stevenson and Miller, 2013; Stevenson et al., 2015).

## Summary and future directions

Given that the neural networks themselves are somehow disrupted in neurodevelopmental disorders, that pathological changes in the dorsal thalamus may be part of altered processes that are taking place throughout the entire network, that neuroimaging has indicated co-occurring changes are seen throughout the reciprocal loop connecting the thalamic relay nuclei with the major cortical multimodal association areas, and critically, that behavioral and cognitive performance is altered as a consequence of subtle manipulations to the MD in animals, it is now, more than ever, relevant to re-evaluate the roles of different subdivisions of the dorsal thalamus in cognitive functions, cognitive control, and neuropsychiatric diseases.

The perspective emerging as an alternative to pure cortico-cortical cognitive processing proposes instead that the higher order thalamic nuclei are closely involved in distributing efference copies of the corticofugal signal to other relevant areas of the cortex (Guillery and Sherman, 2002, 2011; Sherman and Guillery, 2002; Sherman, 2007) and that these transthalamic routes of communication also contribute in an ongoing and rapid manner to helping direct our upcoming responses and reflective knowledge about our internal states/events (Mitchell, 2015). In a sense, it could be conceived of that if the MD nuclei were damaged, or underwent altered neural development, or the interconnections between the MD and cortex were no longer robust then the messages being passed between the cortex (cortico-cortical) and indirectly passed via the thalamus (transthalamic route) may no longer be tightly aligned. Thus our internal representations about what we are currently perceiving (whether this be sights, sounds, tastes, smells, or touch) and representations about what we are doing or about to do (whether this be thinking, deciding, learning, or actually doing) may also be mismatched, and as a result, “noise” of various kinds will occur within the system leading to less optimal performance that may manifest as errors in behavior, or unexplainable thoughts or unusual decisions. Thus, for the brain to function optimally in higher cognitive processes, the MD thalamus is required to support the rapid updating and coordination of cortical messages across interconnected neural networks.

In summary, we have highlighted how different subdivisions of the MD thalamus function as either a key node receiving and then passing on sensory information and related actions to the cortex (lateral MD) or as a key node receiving an efference copy of already processed cortical information from layer 5 corticofugal axons that is transmitted back to other cortical areas via the transthalamic route (MDmc/MDpc/MDcd), with this transmission being managed in the thalamus in an ongoing, dynamic partnership with the cortex, depending on the balance of neuromodulation and inhibitory and excitatory signals. We propose that in addition to the prefrontal cortex and the basal ganglia being the major neural structures involved in cognitive control, that the mediodorsal thalamus (and potentially other higher order nuclei) also be considered critical in allowing optimal cognitive functions to occur.

## Author contributions

ZO and HF: Contributed equally in the writing of this manuscript; All authors contributed to the writing of this review.

### Conflict of interest statement

The authors declare that the research was conducted in the absence of any commercial or financial relationships that could be construed as a potential conflict of interest.
